# MEETINGS CALENDAR - 2017

**Published:** 2017

## June

### ► 1 - Toronto Update on Lung Transplantation and Artificial Lung Support

Toronto, Canada

Informations: Nancy Bush

Phone: 4169782719

E-mail: info-SUR1726@cpdtoronto.ca

Site: www.cpd.utoronto.ca/lungtransplant/

### ► 2 and 3 - Thoracic Refresher

Toronto, Canada

Informations: Nancy Bush

Phone: 4169782719

E-mail: info-SUR1726@cpdtoronto.ca

Site: www.torontothoracicrefresher.ca/

### ► 5 to 9 - Fundamentals in Cardiac Surgery: Part II

Windsor, United Kingdom

Informations: EACTS

E-mail: info@eacts.co.uk

Site: www.eacts.org/academy/2017-programme/

### ► 12 to 14 - Thoracic Surgery: Part II

Windsor, United Kingdom

Informations: EACTS

E-mail: info@eacts.co.uk

Site: www.eacts.org/academy/2017-programme/

### 14 to 17 17th European Congress on Extracorporeal Circulation Technology

Marseille, France

Informations: Judith Moonen

Phone: + 31 6 29 229 655

E-mail: office@fecect.org

Site: www.fecect.org

### ► 15 to 17 - Ventricular Assist Device Co-ordinators Training Course

Berlin, Germany

Informations: EACTS

E-mail: info@eacts.co.uk

Site: www.eacts.org/academy/2017-programme/

### ► 19 to 21 - 18th Annual Cardiologists Conference

Paris, France

Informations: Raul Wilson

Site: annualmeeting.conferenceseries.com/cardiologists/

### ► 20 and 21 - Magna Græcia AORtic Interventional Project® (MAORI) 5th Symposium Complex Diseases of Thoracic and Thoraco-Abdominal Aorta

Catanzaro, Italy

Informations: Prof.Pasquale

Phone: +39 09613695851

E-mail: umgaorta@unicz.it

Site: maori.unicz.it/

### ► 21 to 24 - WTSA 43rd Annual Meeting

Colorado Springs, United States

Informations: Heather Nutter

E-mail: meetings@westernthoracic.org

Site: meetings.westernthoracic.org/

### ► 21 to 24 - ASAIO 63rd Annual Conference

Chicago, United States

Informations: ASAIO Headquarters

Site: www.asaio.com

### ► 28 to 1 - The New Orleans Conference - Las Vegas Edition

Las Vegas, United States

Informations: Joseph Basha

Phone: 318-623-0890

Site: www.TheNewOrleansConference.com

## July

### ► 30 and 1 - Basic Science

Windsor, United Arab Emirates

Informations: EACTS

E-mail: info@eacts.co.uk

Site: www.eacts.org/academy/2017-programme/

### ► 24 and 25 - International Conference on Vascular Biology & Medicine

Chicago, United States

Informations: Suzanne Williams

Site: vascular.cmesociety.com/

